# Bitter Orange *(Citrus aurantium L.)* Intake Before Submaximal Aerobic Exercise Is Safe for Cardiovascular and Autonomic Systems in Healthy Males: A Randomized Trial

**DOI:** 10.3389/fnut.2022.890388

**Published:** 2022-05-27

**Authors:** Cicero Jonas R. Benjamim, Francisco Welington de Sousa Júnior, Andrey Alves Porto, Élida Mara B. Rocha, Milana D. Santana, David M. Garner, Vitor E. Valenti, Carlos Roberto Bueno Júnior

**Affiliations:** ^1^Department of Internal Medicine, Ribeirão Preto Medical School, University of São Paulo, Ribeirão Preto, São Paulo, Brazil; ^2^University Center of the Juazeiro do Norte, Juazeiro do Norte, Brazil; ^3^Autonomic Nervous System Center, São Paulo State University (UNESP), Marilia, São Paulo, Brazil; ^4^Cardiorespiratory Research Group, Department of Biological and Medical Sciences, Faculty of Health and Life Sciences, Oxford Brookes University, Oxford, United Kingdom; ^5^School of Physical Education of Ribeirão Preto, University of São Paulo, Ribeirão Preto, Brazil

**Keywords:** p-synephrine, physical effort, autonomic nervous system, heart rate control and regulation, blood pressure, parasympathetic nervous system

## Abstract

**Background:**

There are still no studies of the cardiovascular safety of the isolated use of *Citrus aurantium* in aerobic submaximal exercise.

**Objective:**

To evaluate the effect of *C. aurantium* supplementation on the recovery of cardiorespiratory and autonomic parameters following a session of submaximal aerobic exercise.

**Methods:**

Twelve healthy male adults achieved a crossover, randomized, double-blind, and placebo-controlled trial. *C. aurantium* (600 mg, p-synephrine at 30% amount [180 mg]) or placebo (600 mg of starch) were ingested 90 min before evaluation in randomized days. We evaluated systolic blood pressure (SBP), diastolic blood pressure (DBP), pulse pressure (PP), mean arterial pressure (MAP), heart rate (HR) and, HR variability indexes at Rest and during 60 min of recovery from exercise.

**Results:**

*Citrus aurantium* ingestion accelerated the reduction in SBP after exercise, anticipated the return of vagal modulation of the heart after exercise via the HF (ms2), pNN50 (%), and 2 UV% indices. Moreover, rushed the output of sympathetic modulation after exercise via the 0V% index. No unfavorable cardiovascular effects were achieved for HR, DBP, PP, and MAP parameters.

**Conclusions:**

*Citrus aurantium* was shown to be safe for the cardiovascular and autonomic systems alongside submaximal aerobic exercise in healthy males.

## Introduction

*Citrus aurantium L*. is a phenylethylamine alkaloid in bitter orange peel, rich in p-synephrine, and abundant in flavonoids (1). p-Synephrine has an adrenergic action and, therefore, the *C. aurantium* is easily applied in weight loss strategies (2) and, thus, contributes to the restoration of hunger and satiety balance regulation of blood glucose, insulin, and triglycerides (3).

P-synephrine has an affinity with β3-adrenergic receptors, seems capable of stimulating lipolysis without compromising cardiovascular activity at rest, unlike other substances (e.g., caffeine, ephedrine) (4). Recently, Guitiérrez-Hellín et al. (5) demonstrated that *C. aurantium* supplementation could elevate fat consumption rates in submaximal aerobic exercise and, therefore, this has made *C. aurantium* a widely used substance to cut the levels of body fat. Nevertheless, there are no assessments of the cardiovascular safety of *C. aurantium* in combination with aerobic submaximal exercise, and evidence regarding its use is rare but needed to guide clinical prescriptions that have *C. aurantium* as a therapeutic option.

In this way, the analysis of cardiorespiratory parameters in combination with autonomic control of heart rate (HR) after physical exercise has been widely enforced to assess cardiovascular risk. Through the scrutiny between heartbeats (RR intervals) or HR variability (HRV), it is possible to study the efferent flow of sympathetic and parasympathetic autonomic to the heart. During exercise, there is a vagal or parasympathetic withdrawal and, then, there is an upsurge in sympathetic modulation to the heart, revealing a surge in HR and cardiac contractility. Upon cessation of exercise, it is expected that there will be a fast reactivation of vagal modulation, which will provide an abrupt reduction and recovery in HR (6). Recent studies have fixated on examining whether nutritional interventions (e.g., energy drinks, caffeine) (7, 8) can delay or accelerate HRV recovery and hence, appreciate its effects on cardiovascular health.

Based on these aforementioned considerations, it was probed as to whether the supplementation of *C. aurantium* prior to aerobic physical exercise could impact the autonomic control of HR and interfere with cardiovascular recovery following exercise. We assume that *C. aurantium* would not affect the recovery of cardiovascular and autonomic parameters after exercise. Given these declarations, we intended to assess the effect of *C. aurantium* supplement on cardiovascular recovery and autonomic constraints after submaximal aerobic exercise.

## Materials and Methods

### Trial Design

This is a randomized study, double-blind, placebo-controlled crossover clinical trial. According to the Declaration of Helsinki, the intervention protocols were approved by the Research Ethics Committee Institutional—Brazil (Process: 26730419.6.0000.5624). The register of study details on Clinicaltrials.gov (https://clinicaltrials.gov/ct2/show/NCT04875143).

### Participants

**Figure 1:**
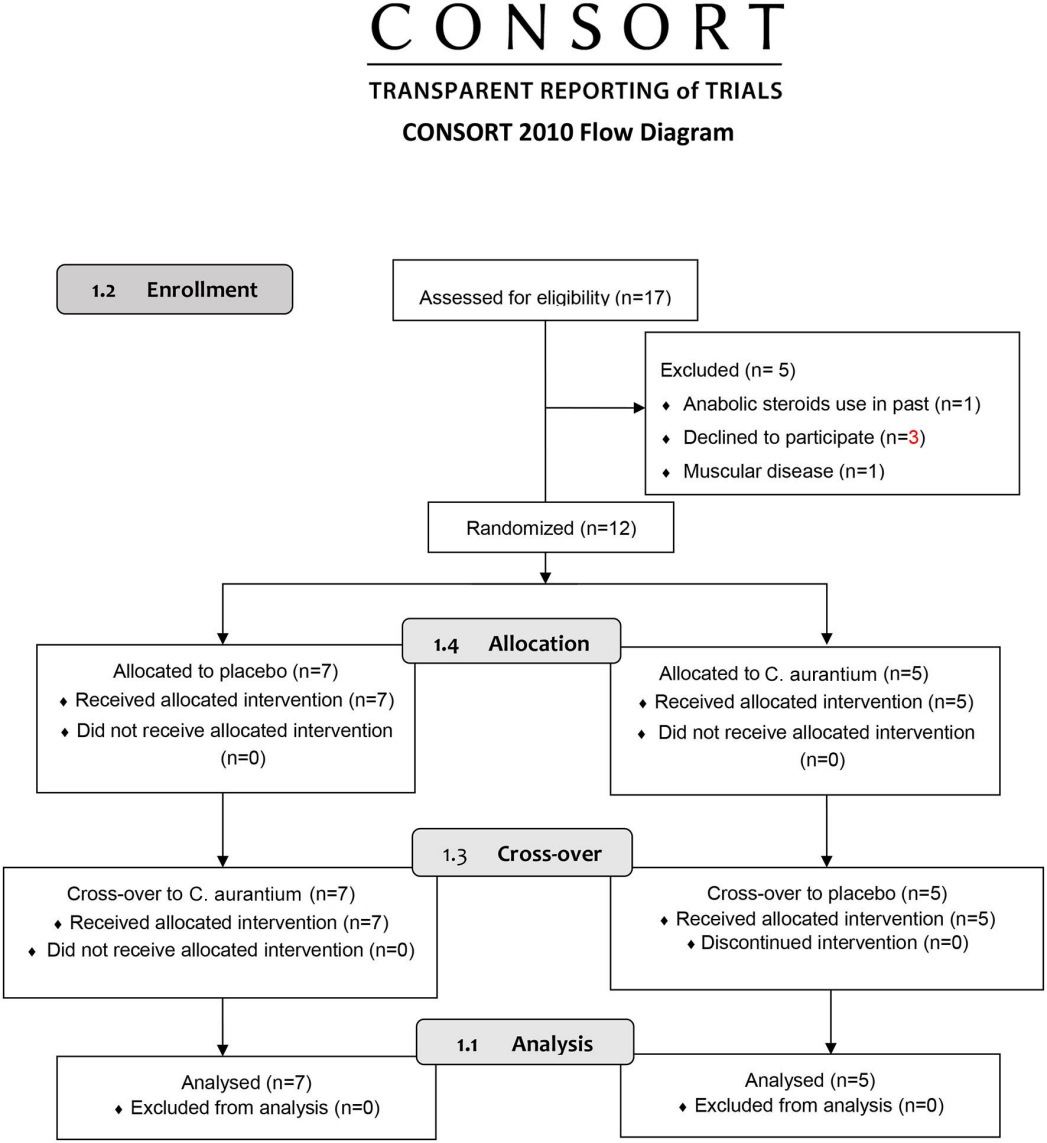
Flow diagram.

We recruited 17 male subjects via social media (e.g., Instagram, Facebook, etc.) to participate in the study. Participants were between 18 and 30 years of age, had a body mass index (BMI) between 18.5 and 29.9 kg/m^2^, and were physically active according to the International Physical Activity Questionnaire (IPAQ) (9). Through screening, we studied the presence of some conditions that would make them ineligible to participate in the study, for instance, smoking, present and past anabolic steroid usage, cardiorespiratory, neurological or musculoskeletal disorders, use of pharmacotherapies that affect the autonomic nervous system. We excluded subjects with the presence of the root mean square of successive differences between normal heartbeats <15 ms. At the end of the study, the sample consisted of 12 subjects (Figure 1).

### Assessment

The characterization of the sample was completed by collecting information such as age (years), mass (kg), height (cm), and BMI (kg/m^2^) consistent with the protocol by Lohman et al. (10). Additionally, baseline values of heart rate (beats per minute), systolic blood pressure (SBP), and diastolic blood pressure (DBP) (mmHg) were logged (Table 1).

**Table 1:** Mean values followed by their respective standard deviations (minimum and maximum) of age, mass, height, BMI, heart rate, SBP and DBP.

**Variables**	**Values**
Age (years)	20.91 ± 1.44 (18–23)
BMI (kg/m^2^)	25.21 ± 2.06 (22.4–28.72)
Height (m)	1.73 ± 0.06 (1.65–1.87)
Mass (kg)	76.08 ± 6.08 (61.4–84)
Heart rate (bpm)	75.80 ± 13.15 (52.91–92.56)
SBP (mmHg)	119.16 ± 2.86 (110–120)
DBP (mmHg)	78.3 ± 3.92 (70–80)

*BMI, body mass index; kg, kilogram; m, meters; bpm, beats per minute; mmHg, millimeter of mercury*.

### Interventions

The intervention protocols were split into three phases, with an interval of 48–72 h between each protocol to provide time for the participants' physical recovery. The interventions were achieved between 10:00 and 14:00 to minimize circadian influences (11) in a noiseless room with temperatures between 22 and 24°C and humidity between 50 and 60% (8). On the first day, an initial interview was completed with the participating candidates in the study. After screening, eligible applicants were provided with a list of guidelines to abstain from certain citrus fruits (mandarin, sweet, and bitter orange), alcoholic and caffeinated beverages, or nutriments (coffee, sports drinks, chewing gum, chocolate), and exercise 24 h prior to the ensuing study sessions. Participants were told to have a light meal (e.g., bread, low-fat cheese, jam, fruit, and natural juice) 2 h before going into the laboratory and wear comfortable and light clothes to permit necessary physical effort.

Through a random sequence, in the second step, participants were randomized to consume a capsule containing 600 mg *C. aurantium* (p-synephrine at 30% amount [180 mg]) or 600 mg starch (placebo) 90 min prior to the procedure. This amount was selected as it is regularly applied in clinical practice (12). In the final stage, the participants received the protocol contrary to the previous one to safeguard the study's crossover. The website https://www.randomizer.org/ was necessary to generate a random sequence of the intervention. An independent researcher who did not participate in the data logging was responsible for randomizing the interventions, choosing the capsules, and assigning them to the investigator. The capsules were opaque and visibly identical; neither the participant nor the investigator could recognize the capsules' contents. The capsules were attained in commercial form from a reliable provider (Florien Fitoativos® Ltd., Piracicaba, SP, Brazil), and their contents were certificated. Naringin and hesperidin concentrations were not analyzed.

On the intervention days, the participants completed the physical exercise on a treadmill for the first 5 min with HR between 50 and 55% of the estimated maximum HR (208 - 0.7 x age) (13) for a “warm-up” and, next, the treadmill speed (Inbrasport ATL 2000, Brazil) was increased to values equivalent to 65–70% of the estimated maximum HR. The participants persisted for 20 min walking at this speed, and at the end of the activity, the participants were once more seated and monitored for an additional 60 min (8).

### Outcomes

#### Blood Pressure

**Figure 2:**
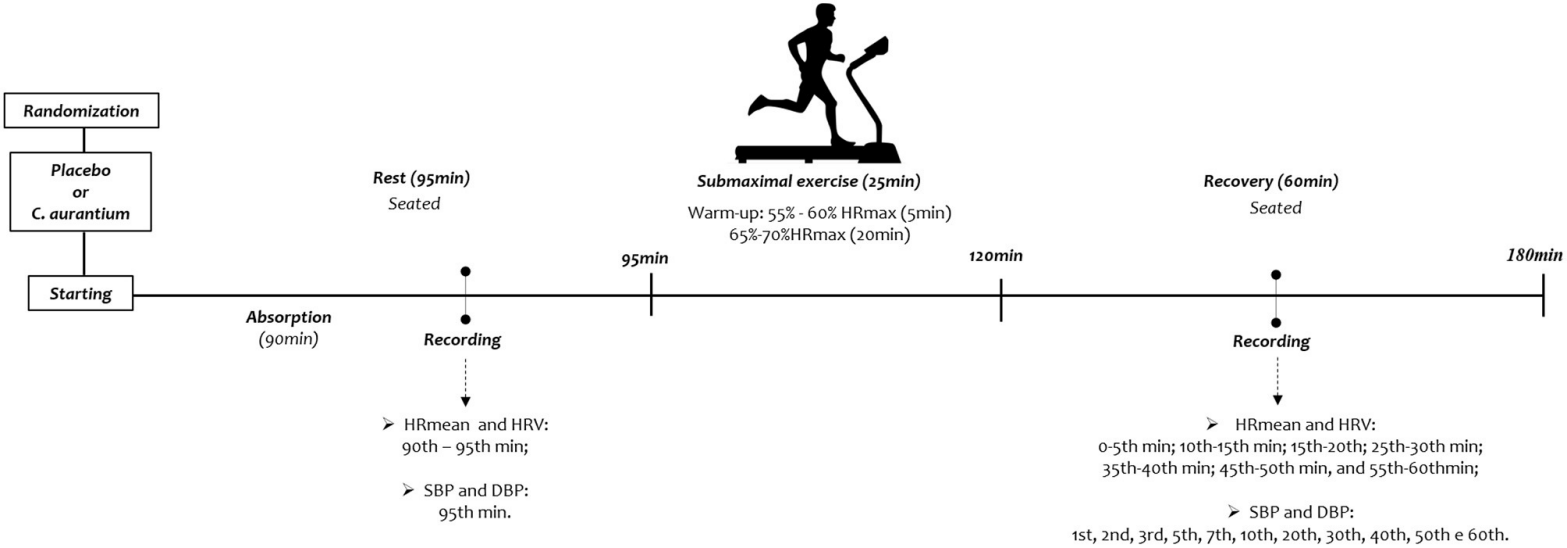
Study design.

BP was logged at Rest−90th to 95th min after capsule ingestion—and throughout recovery - 1st, 2nd, 3rd, 5th, 7th, 10th, 20th, 30th, 40th, 50th, and 60th min after exercise (Figure 2). SBP and BPD measurements were completed indirectly using a stethoscope (Littman Classic II, Saint Paul, USA) and aneroid sphygmomanometer (Welch Allyn Tycos, New York, USA) on the participants' left arm (14). Mean pulse pressure (PP) was considered as the difference between SBP and DBP (PP = SBP - BP) (15). Mean arterial pressure (MAP) was attained by adding one-third of PP to DBP (MAP = 1/3PP + DBP) (16).

#### HR and HRV Recording

For HR and HRV indices scrutiny, cardiac activity was logged beat by beat throughout the data logging technique with a sampling rate of 1 kHz using a Polar® heart rate monitor model RS800CX. The HR and HRV recordings were logged at the following epochs: Rest (R1: 90th to 95th min of resting after capsule ingestion), and all through exercise recovery: 0 to 5th min; 5th to 10th min; 15th to 20th min; 25th to 30th min; 35th to 40th min; 45th to 50th min, and; 55th to 60th min (Figure 2).

Series with 256 regular heartbeats (R-R intervals) were required to make the HRV indices, as recommended by the Task Force of the European Society of Cardiology and the North American Society of Pacing and Electrophysiology (1996). In these series, digital and manual filters were executed to remove artifacts. Only time series with above 95% sinus beats were included in the study (17). After collection, the RR intervals were exported to the software program Kubios® HRV Analysis to produce the linear indices of the frequency domain and time domain (18). Frequency domain analysis was accomplished via spectral analysis by means of the Fast Fourier Transform (FFT) to cause the high frequency (HF) index with a sampling rate of 0.15–0.40 Hz (17). Time-domain analysis was achieved using the percentage of the number of adjacent RR intervals with differences >50 ms (pNN50) (19).

The non-linear HRV analysis was achieved using the PyBios® software (Biomedical Signal Analysis in Python Version 1.2.0 (20) (FMRP/USP, Brazil). We dispersed the number of RR intervals through six levels (0–5), transforming them into a spatial methodology; a sequence of three symbols. All patterns were independently assembled into two clusters, according to the number and type of variation between symbols:

0V corresponds to no variation [three identical symbols, e.g. (2,2,2) or (4,4,4)];

2UV signifies two opposite variations [three symbols form a peak or valley, e.g. (3,5,3) or (4,1,2)].

The occurrence rates of these clusters (0V and 2ULV) were inspected in percentage (%) (21). The 0V% index is demonstrative of cardiac sympathetic modulation, and the 2 UV% is affiliated with cardiac vagal modulation (22).

#### Sample Size

A pilot study conducted with six participants performed the sample size calculation. We applied the root mean square of successive differences between RR intervals in the online software at www.lee.dante.br, which provided the magnitude of the difference. We measured a standard deviation of 12.8 ms, and the extent of the difference was 14.11 ms. The sample size provided was at least 11 individuals per group, with an alpha risk of 5% and a beta risk of 80%. A sample loss of 30% was measured.

### Statistical Analysis

The Shapiro-Wilk statistical test was enforced to assess data normality. For the cardiovascular recovery and autonomic reactivity analysis during the experimental protocols (Rest vs. recovery), One-way analysis of variance (ANOVA1) for repeated measures and the Bonferroni post-test was enforced when the assumption of data normality was attained. Friedman's test followed by Dunn's post-test was required for data that did not acquire a normal distribution. Statistical significance was set at *p* < 0.05 (or, <5%) for all analyses. Cohen's d calculated effect sizes to measure the magnitude of changes for significant differences. We considered >0.9 a large effect size, between <0.9 to >0.5 a medium effect size and, <0.5 a small effect size (23). The confidence interval was calculated considering a probability of 95%. Assessments were achieved using Statistical Package for the Social Sciences (SPSS) (IBM® SPSS Statistics v. 22.0, USA).

## Results

The descriptive data of twelve healthy males that met the study criteria are included in Table 1. These datasets strengthen the homogeneity of our sample.

### Heart Rate and Blood Pressure Following Exercise

The HR recovery analysis revealed no significant differences between the protocols. In the placebo protocol, the comparison of resting and after exercise established an increase in HR from 0 to 5th min of recovery (Rest vs. 0–5 min: Cohen's *d* = 1.84, *p* < 0.001). In the *C. aurantium* protocol, the same results were attained, and HR values remained significantly enlarged from 0 to 5th min of recovery (Rest vs. 0–5min: Cohen's *d* = 1.55, *p* < 0.001) (Table 2).

**Table 2:** Mean heart rate values followed by standard deviations [confidence interval 95%] before and post-exercise in individuals during experimental protocols.

**Variables**	**Treatment**	**Rest**	**Rec (0–5 min)**	**Rec (5–10 min)**	**Rec (15–20 min)**	**Rec (25–30 min)**	**Rec (35–40 min)**	**Rec (45–50 min)**	**Rec (55–60 min)**
HRmean (bpm)	Citrus	75.35 ± 11.9 [68.5–82.1]	90.82 ± 7.49 [86.5–95]^*^	84.56 ± 7.0 [80.5–88.5]	80.89 ± 7.38 [76.7–85]	79.11 ± 9.13 [73.9–84.2]	77.49 ± 7.03 [73.5– 81.4]	76.51 ± 7.13 [72.4– 80.5]	76.67 ± 8.3 [71.9– 81.3]
	Placebo	74.17 ± 9.6 [68.7–79.5]	90.83 ± 8.47 [86–95.6]^*^	84.16 ± 10 [78.5–89.8]	79.90 ± 9.10 [74.7– 85]	78.56 ± 9.20 [73.3– 83.7]	76.97 ± 8.80 [71.9–81.9]	76.21 ± 7.34 [72–80.3]	75.82 ± 8.25 [71.1–80.4]

*Rest, pre-exercise; Rec, Recovery post-exercise; HRmean, mean heart rate during interval recording; bpm, beats per minute*.

* *Difference in relation to rest (ANOVA followed by the Bonferroni post-test, p < 0.05)*.

No significant changes were identified in the *C. aurantium* intervention during the recovery analysis (rest vs. recovery) for DBP, MAP, and PP. Only SBP demonstrated significant changes in 1 min following exercise (Rest vs. 1th: Cohen's *d* = 2.13, *p* < 0.001) in *C. aurantium* protocol. During the placebo protocol, SBP remained significantly higher during 3 min of recovery compared to rest (Rest vs. 1th: Cohen's *d* = 2.50; Rest vs. 3rd: Cohen's *d* = 1.35, *p* < 0.001), we did not observe substantial changes in the values of DBP, MAP, and PP (Table 3).

**Table 3:** Systolic and diastolic blood pressure values followed by standard deviations [confidence interval 95%] before and post-exercise in individuals during experimental protocols.

**Variables**	**Treatment**	**Rest**	**Rec (1 min)**	**Rec (3 min)**	**Rec (5 min)**	**Rec (7 min)**	**Rec (10 min)**	**Rec (20 min)**	**Rec (30 min)**	**Rec (40 min)**	**Rec (50 min)**	**Rec (60 min)**
SBP (mmHg)	Citrus	120.83 ± 4.93 [118.04–123.62]	132.50 ± 5.95^*^ [129.13–135.87]	126.67 ± 9.43 [121.33–132.0]	121.67 ± 7.99 [117.14–126.19]	120.83 ± 8.62 [115.96–125.71]	119.17 ± 6.40 [115.55–122.79]	117.50 ± 4.33 [115.05–119.95]	117.50 ± 4.33 [115.05–119.95]	116.67 ± 4.71 [114–119.33]	116.67 ± 4.71 [114–119.33]	116.67 ± 4.71 [114–119.33]
	Placebo	120.83 ± 6.4 [117.21–124.45]	136.67 ± 6.24^*^ [133.14–140.20]	130 ± 7.07^*^ [126–134]	125.83 ± 4.93 [123–128.62]	123.3 ± 4.71 [120.6–126]	122.5 ± 4.33 [120–124.9]	119.17 ± 2.76 [117.6–120.73]	119.17 ± 2.76 [117.6–120.7]	119.17 ± 2.76 [117.6–120.7]	119.17 ± 2.76 [117.6–120.73]	119.17 ± 2.76 [117.6–120.73]
DBP (mmHg)	Citrus	80.83 ± 4.93 [78.04–83.62]	83.33 ± 6.24 [79.8–86.86]	82.50 ± 5.95 [79.13–85.87]	80.83 ± 4.93 [78.04–83.62]	80.83 ± 4.93 [78–83.62]	80 ± 5.77 [76.73–83.27]	79.17 ± 4.93 [76.38–81.96]	79.17 ± 4.93 [76.38–81.96]	79.17 ± 4.93 [76.38–81.96]	79.17 ± 4.93 [76.38–81.96]	79.17 ± 4.93 [76.38–81.96]
	Placebo	80 ± 0.0 [80–80]	85 ± 10.41 [79.11–90.89]	83.3 ± 7.45 [79.12–87.55]	82.50 ± 5.95 [79.13–85.87]	82.50 ± 5.95 [79.13–85.87]	81.67 ± 5.53 [78.54–84.79]	80 ± 4.08 [77.69–82.31]	80 ± 4.08 [77.69–82.31]	80 ± 4.08 [77.69–82.31]	80 ± 4.08 [77.69–82.31]	80 ± 4.08 [77.69–82.31]
MAP (mmHg)	Citrus	94.17 ± 4.11 [82.41–105.9]	99.72 ± 5.35 [89.62–110.2]	97.22 ± 6.36 [87.62–106.8]	94.44 ± 5.33 [83.93–104.96]	94.17 ± 5.46 [83.65–104.68]	93.06 ± 4.80 [81.30–104.82]	91.94 ± 3.96 [78.37–105.52]	91.94 ± 3.96 [78.37–105.52]	91.67 ± 3.97 [78.09–105.52]	91.67 ± 3.87 [78.09–105.25]	91.67 ± 3.87 [78.09–105.25]
	Placebo	93.61 ± 2.13 [70.05–117.17]	102.22 ± 6.98 [88.62–115.82]	98.89 ± 4.37 [82.23–115.55]	96.94 ± 3.46 [77.71–116.18]	96.11 ± 4.27 [79.45–112.77]	95.28 ± 4.19 [78.62–111.94]	93.06 ± 2.87 [69.50–116.62]	93.06 ± 2.87 [69.50–116.62]	93.06 ± 2.87 [69.50–116.62]	93.06 ± 2.87 [69.50–116.62]	93.06 ± 2.87 [69.50–116.62]
PP (mmHg)	Citrus	40 ± 5.77 [29.48–50.52]	49.17 ± 6.40 [39.56–58.77]	44.17 ± 7.59 [35.28–53.06]	40.83 ± 6.40 [31.23–50.44]	40 ± 7.07 [31.11–48.89]	39.17 ± 7.59 [30.28–48.06]	38.33 ± 5.53 [27.82–48.85]	38.33 ± 5.53 [27.82–48.85]	37.50 ± 5.95 [26.98–48.02]	37.50 ± 5.95 [26.98–48.02]	37.50 ± 5.95 [26.98–48.02]
	Placebo	40.83 ± 6.40 [27.23–54.44]	51.67 ± 12.80 [42.05–61.29]	46.67 ± 12.47 [37.05–56.29]	43.33 ± 9.43 [32.23–54.44]	40.83 ± 7.59 [28.24–53.43]	40.83 ± 6.40 [27.23–54.44]	39.17 ± 4.93 [22.51–55.83]	39.17 ± 4.93 [22.51–55.83]	39.17 ± 4.93 [22.51–55.83]	39.17 ± 4.93 [22.51– 55.83]	39.17 ± 4.93 [22.51–55.83]

*Rest, pre-exercise; Rec, Recovery post-exercise; SBP, systolic blood pressure; DBP, diastolic blood pressure; mmHg, millimeter of mercury*.

* *Difference in relation to rest (Friedman followed by the Dunn's test, p < 0.05)*.

### Heart Rate Variability Recovery After Exercise

**Figure 3:**
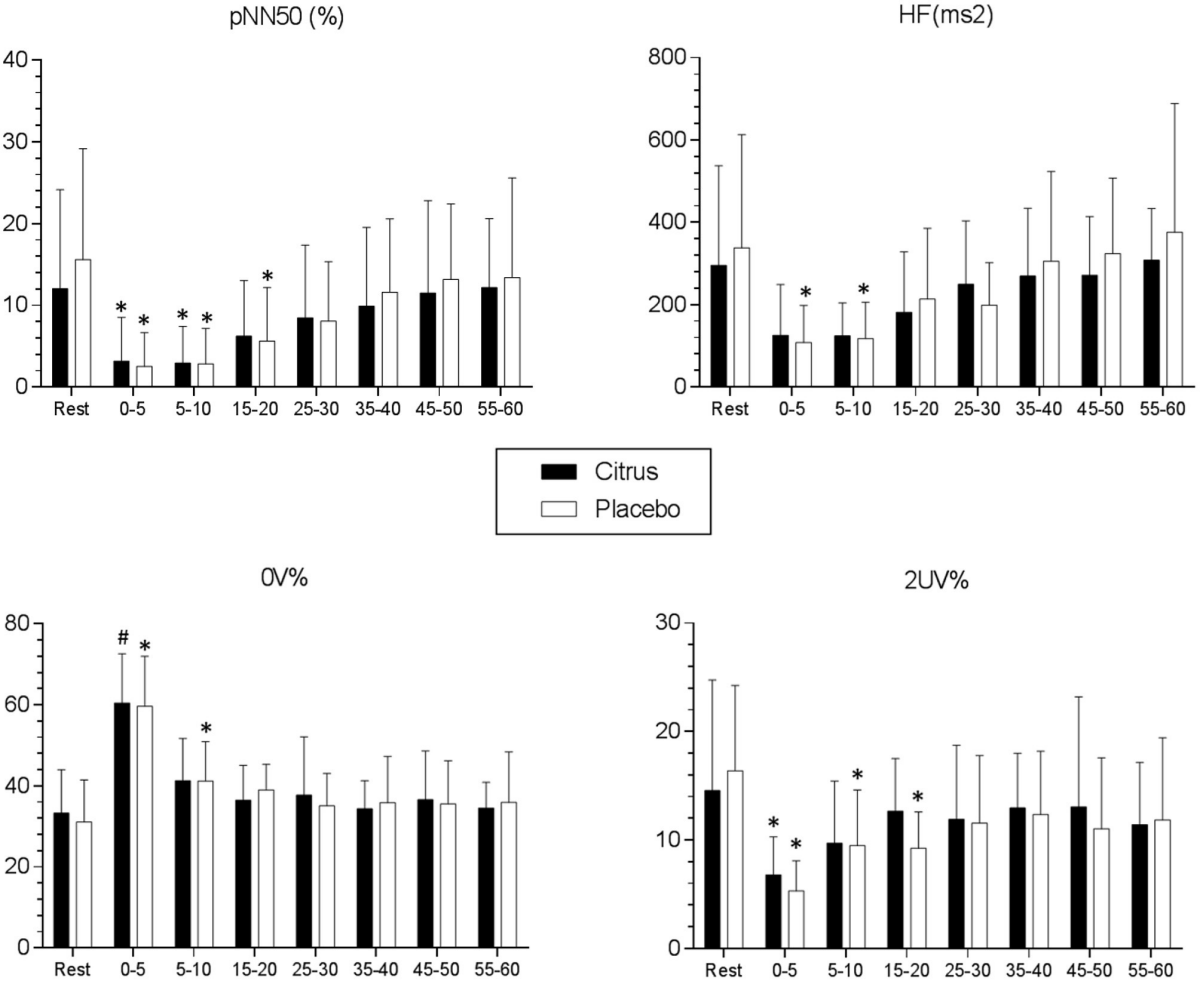
Mean values and respective standard deviations of pNN50 (%), HF (ms^2^), 0V% and 2UV% obtained at rest and during recovery from submaximal aerobic exercise. *Values with significant differences in relation to rest (*p* < 0.05) (Friedman, Dunn's post-test); ^**#**^Values with significant differences in relation to rest (*p* < 0.05) (ANOVA1, Bonferroni's post-test); pNN50 (%): percentage of adjacent R-R intervals with a difference of duration >50 ms; HF (ms^2^): High frequency spectrum (0.15–0.40 Hz); 0V%: zero variations; 2 UV%: two unlike variations.

Time and frequency domain indices in addition to non-linear analyzes revealed that autonomic heart rate recovery occurred more quickly in the *C. aurantium* protocol compared to the placebo protocol. In the placebo protocol, the investigation of recovery (rest vs. recovery) of the HF index revealed that its values remain depressed throughout 10 min of recording after exercise (Rest: 338.25 + 262.8 [CI 95% = 189.5–486.9] vs. 0–5 min: 108.2 + 85.9 [CI 95% = 59.6–156.9] Cohen's *d* = −1.17; Rest vs. 5–10 min: 117.3 + 85.3 [CI 95% = 69–165.6] Cohen's *d* = −1.13, *p* < 0.001). In the *C. aurantium* protocol, vagal return occurred rapidly and the HF index did not attain a significant lessening after exercise (Rest: 295 + 231.9 [CI 95% = 173.4–417.1] vs. 0–5 min: 125.8 + 117.7 [CI 95% = 64.15–187.5], *p* > 0.05; Rest vs. 5–10 min: 124.5 + 76.46 [CI 95% = 83.8–165.1], *p* > 0.05) (Figure 3).

In the placebo protocol, pNN50 index values continued to be significantly decreased throughout 20 min of recovery related to resting values (Rest: 15.6 + 13 [CI 95% = 8.25–22.9] vs. 0–5 min: 2.54 + 3.97 [CI 95% = 0.29–4.78] Cohen's *d* = −1.35; Rest vs. 5–10 min: 2.84 + 4.16 [CI 95% = 0.48–5.19] Cohen's *d* = −1.32; Rest vs. 15–20 min: 5.64 + 6.27 [CI 95% = 2.08–9.19] Cohen's *d* = −0.97, *p* < 0.001). In the *C. aurantium* protocol, pNN50 was demonstrated to be reduced during only 10 min of recovery after exercise (Rest: 12 + 11.5 [CI 95% = 5.98–18.1] vs. 0–5 min: 3.19 + 5.12 [CI 95% = 0.49–5.88] Cohen's *d* = −0.99; Rest vs. 5–10 min: 2.93 + 4.33 [CI 95% = 0.65–5.21] Cohen's *d* = −1.04, *p* < 0.001); Rest vs. 15–20 min: 6.24 + 6.52 [CI 95% = 2.82–9.66], *p* > 0.05 (Figure 3).

The 0V% representative of sympathetic modulation remained higher in recovery from exercise for 10 min in the placebo protocol (Rest: 31.1 + 9.96 [CI 95% = 25.4–36.7] vs. 0–5 min: 59.7 + 11.7 [CI 95% = 53–66.3] Cohen's *d* = 2.62; Rest vs. 5–10 min: 41.2 + 9.27 [CI 95% = 36–46.5] Cohen's *d* = 1.05, *p* < 0.001). In the *C. aurantium* protocol, the 0V% index values reduced quicker and significant differences compared to rest were only identified during 5 min after exercise (Rest: 33.2 + 10.3 [CI 95% = 26.9–39.6] vs. 0–5 min: 60.5 + 11.6 [CI 95% = 51.1–69.8] Cohen's *d* = 2.48, *p* < 0.001; Rest vs. 5–10 min: 41.2 + 10 [CI 95% = 34.3–48.2], *p* > 0.05 (Figure 3).

The 2 UV% index typical of parasympathetic modulation remained lessened in the placebo protocol for a period of 20 min after cessation of physical exercise (Rest: 16.4 + 7.52 [CI 95% = 12.1–20.6] vs. 0–5 min: 5.32 + 2.65 [CI 95% = 3.81–6.82] Cohen's *d* = −1.96; Rest vs. 5–10 min: 9.48 + 4.91 [CI 95% = 6.69–12.2] Cohen's *d* = −1.08; Rest vs. 15–20 min: 9.25 + 3.19 [CI 95% = 7.43–11] Cohen's *d* = −1.23, *p* < 0.001). In the *C. aurantium* protocol, there was a substantial reduction of the 2 UV% index only in the first 5 min of recovery after exercise (Rest: 14.5 + 9.75 [CI 95% = 9.4–19.7] vs. 0–5 min: 6.78 + 3.35 [CI 95% = 4.95–8.61] Cohen's *d* = −1.06, *p* = 0.002; Rest vs. 5–10 min: 9.71 + 5.46 [CI 95% = 6.78–12.6], *p* > 0.05; Rest vs. 15–20 min: 12.6 + 4.64 [95%CI = 9.96–15.3], *p* > 0.05) (Figure 3).

## Discussion

Our findings demonstrate that the ingestion of *C. aurantium* (p-synephrine 180 mg) prior to exercise fast-tracks the fall in SBP after physical exertion. In *C. aurantium* protocol, the decreased sympathetic modulation (0V%) to the heart after exercise occurred rapidly, moreover, the return of HR vagal control through recovery after exercise is intensified from the HF (ms2), pNN50 (%), and 2UV% indexes that represent the parasympathetic control of the cardiac rhythm.

Earlier studies propose that one of the benefits of using *C. aurantium* equated to other adrenergic substances (e.g., caffeine) is its selectivity in binding to β-3 adrenergic receptors. Activation of β-3 adrenergic receptors triggers reverse inotropic effects, antagonizing the activation of further classes of adrenoreceptors (β-1 and β-2) in cardiac tissue and, thus, decreasing sympathetic modulation to the heart. This clarifies why overall, the binding of p-synephrine with β-3 adrenergic receptors does not increase BP or HR, displaying cardioprotective effects (24).

In this study, in the placebo intervention, for the spectral analysis, the HF index, representative of parasympathetic modulation, needed 10 min after termination of exercise to recover. In the *C. aurantium* protocol, we did not find substantial changes in the HF index in exercise recovery vs. rest. Analogous deviations occurred in the pNN50 index and were reduced 20 min after cessation of exercise in the placebo protocol. While in the protocol with *C. aurantium*, this index continued to be reduced for only 10 min after exercise.

In the placebo protocol, the non-linear analysis of HRV exposed that the values of 0V% (sympathetic modulation) remained high after 10 min of exercise cessation, in the *C. aurantium* protocol, transformations were only following 5 min of recovery. The values of the 2 UV% index (parasympathetic modulation) continued to be reduced for 20 min in the placebo protocol. However, in the *C. aurantium* protocol, the values were only meaningfully reduced for 5 min after the cessation of exercise.

These observations make *C. aurantium* a safe nutritional compound to be applied during exercise, which supports the recovery of autonomic parameters following exercise. Since a slow post-exercise autonomic recovery is linked with an increased cardiovascular risk (25), the results of our study indicate that *C. aurantium* compounds have a potential preventive role on the onset of cardiovascular complications in physical exercise.

As caffeine and *C. aurantium* are frequently sold as complementary formulas for use in humans, preceding studies have assessed the effects of using these substances alone and in combination. Through a randomized clinical trial, Guitiérrez-Hellín et al. (5) assessed whether using *C. aurantium* alone or in combination with caffeine would have different results for fat utilization during aerobic physical exercise. No superiority was found between *C. aurantium* alone and combined with caffeine on the total values of fat consumption during the physical exercise session, while both interventions were superior to the placebo treatment.

This supports the isolated use of *C. aurantium* an alternate way to be applied as an adjunct in cutting body fat without inducing cardiac risk. In the study by Guitiérrez-Hellín et al. (5), HR and SBP values were not altered with *C. aurantium* isolated supplement. In contrast, the HR and SBP were significantly higher when caffeine was included in the formulation. Our study achieved no changes for HR, and SBP was lessened more quickly following exercise.

The identification of β-3 adrenoreceptors in cardiovascular tissues posed challenges to the paradigm of sympathetic regulation by β-1 and β-2 adrenoceptors. The binding response of p-synephrine to the β-3 receptor may elucidate why no increase in HR or BP is detected when *C. aurantium* is enforced alone. In contrast, when *C. aurantium* is combined with caffeine in dietary supplements, it is capable of affecting these parameters, particularly in caffeine-sensitive individuals (26). It has been revealed that the combination of these substances promotes a significant increase in the concentration of plasma catecholamines (e.g., epinephrine and norepinephrine), which increases the sympathetic nervous systems' activity (4).

The study by Kliszczewicz et al. (26) revealed that the combined use of caffeine and *C. aurantium* upsurges sympathetic modulation to the heart throughout rest and corroborates the increases in HR and SBP achieved in the study by Guitiérrez-Hellín et al. (5). It is assumed that caffeine alone can increase HR during physical exercise (27). Despite that, a recent meta-analysis demonstrated that caffeine could not delay vagal return to the heart after exercise, evaluated by the HF and root mean square of successive differences between RR intervals (RMSSD) indices (28). Equally, Kliszczewicz et al. (26) detected no differences in HRV recovery after exercise with caffeine and *C. aurantium* combined.

Caffeine and *C. aurantium* combination have no extra effects on exercise fat utilization (5). These substances appear to exhibit the opposite cardiovascular effects and, thus, caffeine seems to overlap the beneficial effects of the isolated use of *C. aurantium* on cardiovascular health. In this study, *C. aurantium* supplementation alone optimized the recovery of SBP and HRV indices after exercise. The nutritional characteristics demonstrated in the flavonoids (e.g., naringenin and hesperetin) of *C. aurantium* perform antioxidant and anti-inflammatory activities, which are partly answerable for accelerating the return of parasympathetic control of heart rate seen by vagal indices of HRV. Such properties can hasten the removal of metabolites produced by physical exercise, restoring baroreflex sensitivity and decreasing metaboreflex activation more quickly at the end of physical exercise (21).

While *C. aurantium* exhibited cardioprotective effects, it is essential to be careful with its usage. Bui et al. (29) demonstrated that a dose of 900 mg (6% of p-synephrine) increases HR, SBP, and DBP for up to 5 h after ingestion. Yet, in other studies that enforced doses beneath 600 mg in an acute (5, 30, 31) and chronic (for 15 days) (32) form, no changes were achieved for the HR, SBP, and DBP values, nor electrocardiographic disturbances. Likewise, our results do not support the findings of Bui et al. (29). The results from the study of Ratamess et al. (33) strengthen our findings. In your results, the p-synephrine supplementation (100 mg) did not evoke changes in HR before, during, and following resistance exercise unless 100 mg of caffeine was added to the formulation. The same occur in the rest situation, in another study by Ratamess et al. (30), an intake of 103 mg of p-synephrine not elevated HR or SBP, but when 337 mg of caffeine were added to the formulation HR and SBP were higher.

The study of Bui et al. (29) presented some faults in methodology. Although it is a randomized and crossover study, there is a lack of information about allocation order in the study. The “light” breakfast offered 1 h after bitter orange supplementation could be mitigated the effects of C. aurantium, and provoked adjustments in blood pressure, because of higher sweet and fat content (e.g., cream cheese, bagels, and milk) (34). Furthermore, the authors did not report guarantees that snack was equal on the others evaluation days. Bitter orange caused cardiovascular effect was only observed based on statistical adjustments. A difference was seen compared to placebo but not when compared to baseline. All these factors raise questions about the validity of their conclusions.

The results recognized in our analyses will advance health professionals' conduct who work with the prescription of nutritional supplements. *C. aurantium* (600 mg with 30% p-synephrine) is safe to use in combination with submaximal aerobic exercise in a healthy population. Consequently, it may be an alternative way to replace other compounds that demonstrate similar contributions regarding fat utilization during exercise but that promote unwanted cardiovascular effects (e.g., caffeine) (27).

Our study highlights important points about the study population, given that it is restricted to healthy and physically active males. Notwithstanding the number of participants having exceeded the sample size calculation, the final sample is considered small. An important detail regarding the consequences is that the effect sizes achieved were mostly large (>0.9) for the statistical differences between the intervention protocols in analyzing the recovery of HRV indices in response to exercise, which supports our results.

Eight (66%) participants had a BMI > 24.9 kg/m^2^ and, therefore, most of the population in this study is overweight, which increases the external validity of our findings as overweight individuals predominantly resort to this type of intervention. With the desire to improve body composition. In spite of this, these facts do not allow these results to be extrapolated to other populations and, therefore, further research with obese individuals is needed to confirm the safety of using *C. aurantium* in combination with exercise. For the time being, we prefer to use a healthy population free from metabolic disorders to prevent possible adverse events from *C. aurantium* supplementation. Nevertheless, we encourage further studies to be established with *C. aurantium* as an intervention with these preliminary data. Studies with females and other health conditions should also be performed to increase the external validity of these data and expand the application of *C. aurantium*.

## Conclusion

*C. aurantium* promoted the resumption of parasympathetic control and output of sympathetic flow of cardiac rhythm after physical exercise and decreased SBP. Based on these and previous findings, we assume that *C. aurantium* is a safe nutritional compound with submaximal aerobic exercise in healthy males when used appropriately, moreover, your combination with a good diet there could be improved fat oxidation in exercise without the cardiovascular risk.

## Data Availability Statement

The raw data supporting the conclusions of this article will be made available by the authors, without undue reservation.

## Ethics Statement

The studies involving human participants were reviewed and approved by University Center of the Juazeiro do Norte (Process: 26730419.6.0000.5624 – December 18th, 2019). The patients/participants provided their written informed consent to participate in this study.

## Author Contributions

CJRB supervised the study, performed experiments, performed the statistical analysis, wrote the introduction, methods, discussion, and results in sections. FJ, ER, and MS collected data and performed conduction of experiments. AP performed the statistical analysis, improved interpretation analysis, and wrote the results in sections. DG drafted the manuscript, improved interpretation analysis, and reviewed English grammar and spelling. VV and CRBJ supervised the study, reviewed the manuscript content, and gave final approval for the version submitted for publication. All authors contributed to the article and approved the submitted version.

## Conflict of Interest

The authors declare that the research was conducted in the absence of any commercial or financial relationships that could be construed as a potential conflict of interest.

## Publisher's Note

All claims expressed in this article are solely those of the authors and do not necessarily represent those of their affiliated organizations, or those of the publisher, the editors and the reviewers. Any product that may be evaluated in this article, or claim that may be made by its manufacturer, is not guaranteed or endorsed by the publisher.
